# Optimisation and Validation of a Quantitative Method for the Analysis of Polymers of Nanoplastics in Human Faeces

**DOI:** 10.3390/molecules31111947

**Published:** 2026-06-04

**Authors:** Eloy Torres, Mireia Obon, Víctor Moreno, Ferran Moratalla-Navarro, Jordi Esquena, Marta Llorca, Marinella Farré

**Affiliations:** 1 ON-HEALTH Group, Institute for Environmental Assessment and Water Research (IDAEA-CSIC), 08034 Barcelona, Spain; eloy.torres@idaea.csic.es; 2 ONCOBELL Program, Bellvitge Biomedical Research Institute (IDIBELL), 08908 L’Hospitalet de Llobregat, Barcelona, Spain; mireiaobon@iconcologia.net (M.O.); v.moreno@iconcologia.net (V.M.);; 3 Unit of Biomarkers and Susceptibility (UBS), Oncology Data Analytics Program (ODAP), Catalan Institute of Oncology (ICO), 08908 L’Hospitalet del Llobregat, Barcelona, Spain; 4 Consortium for Biomedical Research in Epidemiology and Public Health (CIBERESP), 28029 Madrid, Spain; 5 Department of Clinical Sciences, Faculty of Medicine, and Health Sciences and Universitat de Barcelona Institute of Complex Systems (UBICS), University of Barcelona (UB), 08908 L’Hospitalet de Llobregat, Barcelona, Spain; 6 Institute for Advanced Chemistry of Catalonia (IQAC-CSIC), and Networking Research Center on Bioengineering, Biomaterials and Nanomedicine (CIBER-BBN), 08034 Barcelona, Spain; jordi.esquena@iqac.csic.es

**Keywords:** micro- and nanoplastics, human faeces, high-resolution mass spectrometry

## Abstract

Concerns about human exposure to micro- and nanoplastics (MNPLs), particularly nanoplastics (NPLs), have intensified in recent years. Consequently, there is a growing need for validated quantitative analytical methods capable of assessing NPLs in complex human biological matrices. Current approaches for NPL analysis are still limited by the absence of standardised protocols, difficulties in avoiding background contamination, and challenges associated with the selective identification and quantification of polymer-specific nanoparticles. Moreover, most common approaches for quantification by particle counting cannot be applied for NPLs < 500 nm. In this study, we developed and validated an analytical method for the detection and quantification of NPLs in human faeces. As an initial step, polyethylene (PE) and polypropylene (PP) nanoparticles (NPs) were synthesised using bottom-up methods and characterised by dynamic light scattering (DLS) and electron microscopy (SEM and TEM). To optimise and assess the extraction, synthetic faeces were prepared and used in spiking experiments to avoid background contamination from plastics. Two digestion strategies were evaluated: (i) Fenton’s reagent followed by strong acid digestion, and (ii) alkaline digestion. Quantitative determination of polymer-specific NPLs was performed by size-exclusion liquid chromatography coupled with high-resolution mass spectrometry and atmospheric-pressure photoionization (SEC-APPI-HRMS). Polymer identification was based on characteristic monomer-loss patterns and Kendrick Mass Defect analysis. Fenton-based digestion showed superior performance, yielding recoveries about 55–66% for PE and 59–61% for PP. The validated method achieved limits of detection and quantification of 0.015 and 0.058 μg/kg for PE, and 0.025 and 0.083 μg/kg for PP, respectively. Precision, expressed as %RSD, was 10.1% for PE and 20.1% for PP. These results demonstrate that SEC-APPI-HRMS combined with Fenton-based digestion provides a sensitive and reliable approach for the quantification of polymer-specific NPLs in human faeces. The method represents an important advance for human biomonitoring studies and supports future research aimed at assessing human exposure and the potential health risks associated with nanoplastics.

## 1. Introduction

The increasing success of plastics from the Second World War to the present is based on their mouldability and lightness, as well as their ability to be produced with a wide range of properties, durability, low cost, and lower energy requirements for production and transport compared to alternative materials like metal or glass [1]. For these reasons, plastics are ubiquitous in our daily lives, used in packaging, clothing, vehicles, and healthcare, but they have become major pollutants.

The impact of plastic pollution is partially influenced by the size of plastic items or particles.

Microplastics (MPLs) are considered plastic pieces below 5 mm, including plastic particles in the nanorange, denoted as nanoplastics (NPLs). MNPLs can have their origin in cosmetics and cleansing products, cloth fibres and tyre erosion [2] (entering the environment at that size and classified as primary MNPLs), and in the environmental fragmentation and erosion of plastic pieces and debris [3] (classified as secondary MNPLs). Humans are exposed to MNPLs through three main routes: ingestion, inhalation, and, to a lesser extent, dermal contact. MPLs, in particular NPLs, can cause physical damage [4], such as inflammatory lesions and oxidative stress, arising from their potential to interact with biological tissues, thereby increasing translocation [5] and uptake. The inability of the immune system to remove synthetic particles may lead to chronic inflammation and increase the risk of neoplasia [6,7]. Several studies reported the presence of MPLs in human matrices, for example, in lung tissues [8], in sputum [9] from patients with respiratory diseases, in human placenta [10], in blood [11], in human colectomy specimens [12], and in human faeces [13,14,15,16,17]. However, to date, most studies have assessed MPLs using spectroscopic techniques, such as Fourier-transform infrared (FTIR) and Raman microscopy, often combined with particle counting approaches [14]. While these methods provide useful information on particle size, shape, and polymer composition, they cannot detect NPLs and are limited in delivering quantitative data in complex matrices [15]. In this regard, it should be highlighted that chromatographic approaches such as pyrolysis–gas chromatography–mass spectrometry (Py-GC-MS) [18] or Py-GC-HRMS are not affected by the particle size, but the limit of detection of this technique restricts its application for biological analytical samples with low NPL contents and complex matrices. Size-exclusion liquid chromatography coupled with high-resolution mass spectrometry (LC-HRMS) offers superior sensitivity and specificity for the detection and characterisation of polymer degradation products, providing valuable chemical insights that complement particle-based techniques.

To protect human health from the new causes of immune disorders, obesity, neurodegenerative diseases, and cancer, the evaluation of NPLs is urgently needed. In this context, the analysis of NPLs in human faeces constitutes a crucial non-invasive approach to assess real-world exposure levels. However, this task presents significant analytical challenges due to the complexity of the matrix, the lack of reference materials and the wide range of particle sizes and polymers diversity. Considering these analytical challenges, the main objective of this work was to develop and validate a robust, sensitive, and reproducible analytical method for the identification and quantification of polyethylene (PE) and polypropylene (PP) NPLs in human faeces.

To achieve this objective, polymer standards in the nanometric range were required. While PP-NPLs are commercially available, suitable PE-NPL standards (~50 nm) are not. Therefore, a first specific objective was the bottom-up synthesis of PE-NPLs, followed by their physicochemical characterisation using scanning electron microscopy (SEM) and particle size distribution analysis by nanoparticle tracking analysis (NTA). These standards were subsequently used to spike synthetic faeces, which were prepared specifically to avoid background plastic contamination from previously used materials. The synthetic faeces were fortified at three concentration levels to evaluate extraction recoveries, as well as the method limit of detection (MLOD) and method limit of quantification (MLOQ) of the optimised analytical procedure. Finally, as a proof of concept, the method was applied to five real samples. To the best of the authors’ knowledge, this study represents the first validation of a quantitative mass-based analytical method for the determination of NPL polymers in human faeces.

## 2. Results and Discussion

### 2.1. Generation of PE-NPs and Characterisation of Polymeric NPs

Two different methods were evaluated for the generation of PE-NPs: Tanaka’s solvent–antisolvent precipitation approach [19] and Merdy’s method based on a water-in-toluene emulsions [20] (both methods are described in Section 2.2). In the first method, PE was dissolved in toluene under reflux and injected into DMSO under magnetic stirring; DMSO acted as an antisolvent, inducing NPs formation. In the second method, PE was first dissolved in toluene, then toluene-in-water emulsions were formed and finally PE was precipitated within the emulsion droplets. In both protocols, the resulting suspensions were purified by dialysis to remove residual solvents, surfactants, and salts, yielding aqueous dispersions of PE-NPs. A schematic overview of both preparation routes is provided in the Supplementary Materials (Figures S1 and S2).

**Figure 1:**
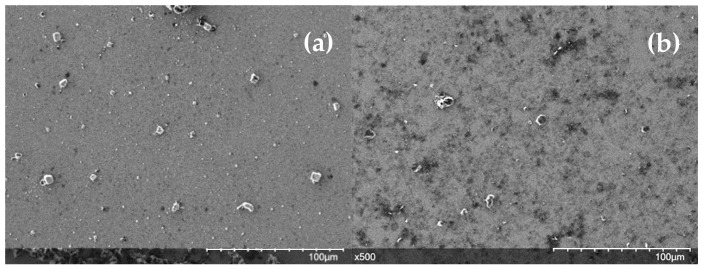
SEM images of microparticle suspensions prepared by Tanaka’s precipitation method, at PE 136 ppm (**a**) and 2324 ppm (**b**) PE concentrations. Scale bars indicate 100 μm.

**Figure 2:**
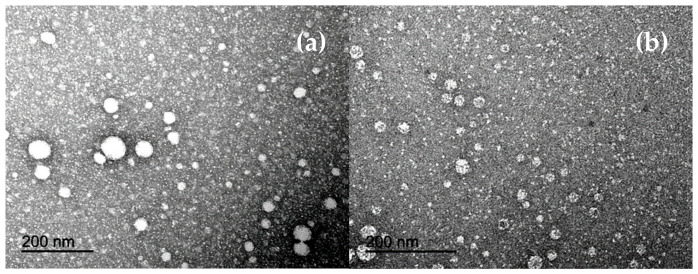
TEM images of the NPs obtained using Merdy’s emulsion method, at 136 ppm (**a**) and 500 ppm (**b**) PE concentrations. Scale bars indicate 200 nm.

The morphology of the NPs was evaluated by SEM. The solvent–antisolvent precipitation method led to large and aggregated particles, producing irregular flocs visible even at low SEM magnification (Figure 1), whereas Merdy’s emulsion–precipitation method produced much smaller, well-dispersed, roughly spherical NPs averaging ~50 nm in diameter, observed by TEM (Figure 2). These differences can be attributed to the use of a surfactant in Merdy’s emulsion method, which promotes emulsification and remains adsorbed on the particle surface, thus providing colloidal stability. In contrast, the solvent–antisolvent precipitation process was carried out without surfactant, leading to particle aggregation in aqueous dispersions due to the strong hydrophobicity of polyethylene and the absence of surface charges capable of providing electrostatic stabilisation. Consequently, the emulsion method was selected for the present study.

Additional TEM images at various polymer concentrations are provided in the Supplementary Materials. Results confirmed that PE particles prepared by solvent–antisolvent precipitation were highly aggregated (Figure S3), whereas PE nanoparticles obtained from O/W emulsions were significantly smaller and not aggregated, although they were polydisperse (Figure S4).

DLS measurements are consistent to these findings (Table 1). Suspensions obtained using Merdy’s method (500 and 136 mg/L) exhibited mean hydrodynamic diameters of 340 ± 61 nm and 259 ± 66 nm, with high polydispersity indices (PDIs) of 0.35 and 0.36, respectively. In contrast, the Tanaka method (39 mg/L) produced larger particles (390 ± 50 nm) with a higher PDI (0.47), indicative of a broader size distribution, probably caused by particle aggregation. In any case, the DLS data should be treated with extreme caution, since high polydispersity leads to inaccurate results. DLS provides an intensity-weighted size distribution, whereas TEM image analysis produces a number-based distribution. Since the scattering intensity is proportional to the sixth power of particle diameter (I ∝ d6), larger particles disproportionately dominate the DLS signal, making the technique highly sensitive to polydispersity and biassed toward larger sizes. In addition, DLS measures the hydrodynamic diameter, which includes not only the nanoparticles but also the adsorbed surfactant layer and the surrounding hydration shell. Consequently, particle sizes determined by DLS are typically larger than those obtained from TEM image analysis.

Regarding the selection of the most suitable synthesis method, NPs produced using the approach described by Merdy et al. exhibited more favourable characteristics, based on SEM, TEM and DLS analysis. Accordingly, this method was selected for further work. Specifically, the 136 mg/L suspension was chosen, as it yielded the most stable NPL dispersion, with a significantly lower degree of aggregation, and individual particles averaging approximately 50 nm in diameter, as determined by TEM.

### 2.2. Optimisation of the Extraction Method for NPLs from Human Faeces

The analytical conditions for the analysis of PE and PP by LC(SEC)-APPI(-)-HRMS were already validated [21] and details are presented in Section 3.6. About organic material elimination, two of the most common approaches for the analysis of plastic particles were assessed. Their performance for separation and organic removal has been previously reported [13,22,23,24,25]. Here we tested which one was more suitable followed by LC(SEC)-APPI(-)-HRMS.

**Alkaline treatment with 10% KOH**: Very briefly, 50 mL of 10% KOH solution were added to 0.5 g of lyophilized sample and the mixture incubated overnight at 60 °C. The following day, 20 mL of a hypersaline NaCl solution was added to the digestate. The mixture was then manually agitated and left to settle for 2 h to promote density-based separation. The supernatant was filtered through 0.7 µm glass microfiber filters. Unlike the Fenton protocol, no acid treatment was applied. The filters were then placed in an oven and dried at 60 °C. Filter extraction and solvent evaporation were carried out following the same procedure described for the Fenton protocol. The final extracts were also kept at −20 °C before injection.

To optimise and validate a quantitative analytical method for assessing polymer content in human faeces, an in-house reference material was employed to evaluate the performance of two extraction protocols by spiking the material at 143 µg/kg.

**Figure 3:**
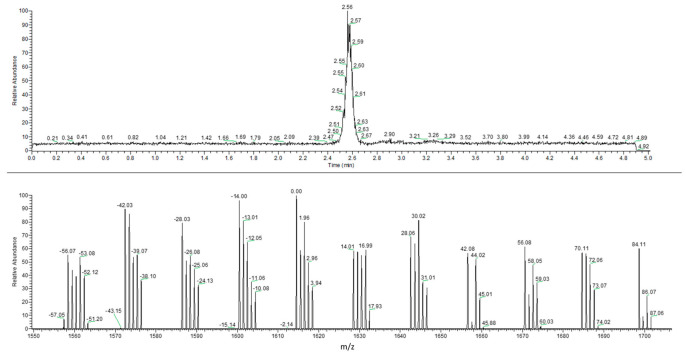
Ion chromatogram and mass spectrum of a PE-spiked sample.

Figure 3 shows the extracted ion chromatogram and mass spectrum for a PE sample, where the monomer losses are clearly observed.

**Figure 4:**
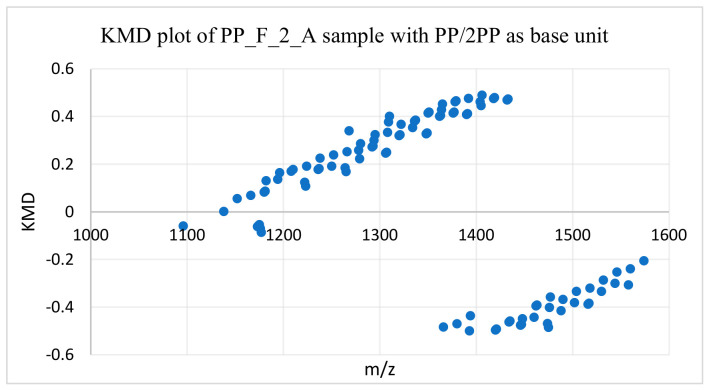
KMD plot reveals two homologous series of polypropylene oligomers.

From the obtained spectra list, KMD analysis was performed, which enabled the identification of polymers by highlighting repeating units in their mass spectra. This analysis was particularly important because both polymers exhibit similar loss patterns related to their monomer units. In Figure 4 a KMD plot for a spiked PP sample is showed.

Matrix effect (%) was calculated according to Equation (1):

(1) $$\mathrm{Matrix}\;\mathrm{effect}\;(\%)\;=\;\frac{Peak\;area\;in\;matrix\;extract}{Peak\;area\;in\;solvent}\;\times \;100$$

The Fenton digestion method exhibited matrix effects, signal enhancement for PP (+31%) and an ion suppression for PE (−52%). The ion suppression observed for the alkaline treatment could lead to the non-detection of the polymers, especially at low concentrations. However, this was compensated by using procedural blanks and matrix-matched calibration curves. Therefore, the Fenton method proved more reliable and suitable for accurate quantification in our case.

### 2.3. Validation of Fenton’s Protocol for the Extraction of MNPLs from Human Faeces

Once the Fenton method was selected, new samples were prepared and spiked at concentrations ranging from 1.4 to 71.4 µg/kg and treated as described in Section 3.5. The quality parameters of the method as well as the recoveries are shown in Table 2.

Linearity was evaluated using external calibration curves prepared with eight concentration levels ranging from 50 to 0.05 μg/L through serial dilution, showing good correlation coefficients (R^2^ ≥ 0.93) for both PP and PE. Intra-day repeatability was assessed by analysing three replicates of spiked samples at 71, 14, and 1.4 µg/L within the same day. The relative standard deviation (RSD) values obtained were between 8.6 and 20.1 for PP, and 7.8 and 24.7% for PE, indicating enough precision. Method limits of detection (MLOD) and quantification (MLOQ) were calculated, with MLODs of 0.75 μg/kg for PP and 0.34 μg/kg for PE, while MLOQs were 2.27 μg/kg for PP and 1.03 μg/kg for PE. Method accuracy was evaluated by recovery experiments, spiking blank samples at various concentrations within the calibration range. Recoveries ranged from 39 to 61% for PP and 55 to 66% for PE, demonstrating acceptable accuracy for both polymers.

### 2.4. Analysis of Real Samples

As a proof of concept, the method was applied to five real faecal samples. Samples were collected in stainless steel trays to avoid cross-contamination and transported to the laboratory within two hours. Upon arrival, fresh stool samples were homogenised in a blender, frozen, and lyophilized. For each sample, 0.5 g of the ground, lyophilized material was treated with the Fenton reagent as described above; extracts were analysed by LC(SEC)-APPI (-)-HRMS. As shown in Table 3, none of the samples contained PP, while two of the five contained relatively high concentrations of PE (34–269 mg/kg dry weight). An extracted ion chromatogram for a real sample is presented in Figure S5 (Supplementary Materials). Although the small sample size prevents firm conclusions, these results are consistent with previous studies reporting a higher frequency and concentration of PE relative to other polymers, including PP.

## 3. Materials and Methods

### 3.1. Reagents and Materials

All the reagents were of HPLC analytical grade for the analysis. Toluene > 99.7% purity CHROMASOLV^®^PLUS, which was used as a mobile phase during HPLC MNPLs-polymer analyses, was acquired from Merck (Darmstadt, Germany), whereas methanol and water were purchased from Fischer Chemical (Loughborough, UK), and dimethyl sulfoxide (DMSO) was purchased from Merck. Glass fibre filters GF/F (pore size of 700 nm) were obtained from Whatman PLC (Maidstone, UK), and nitrogen used as drying gas with 99.995% purity was supplied by Air Liquide (Barcelona, Spain). Dialysis bags (avg. flat width 25 mm, MWCO = 14k Da) were purchased from Merck. The PE analytical standard (M.W. ∼2000 Da) was supplied by Agilent Technologies (Santa Clara, CA, USA) while polypropylene (PP) (M.W. ∼1220 Da) (700 nm diameter) was obtained from American Polymer Standards Corporation (Mentor, OH, USA). The PE standard was used as raw material for producing nanomaterials.

PE and PP individual stock standard solutions were prepared in toluene to achieve a concentration of 1000 mg/L and subsequently used for the calibration curves. Standards were previously solubilized in toluene using a heat plate at 60 °C with constant motion for 2 h. For each individual polymer, initial calibration curves ranged from 0.01 μg/L to 5 mg/L. To prepare each concentration of the calibration curve, an ultrasonic bath was used for 5 min after each dilution step to ensure homogenization. All concentrations were vortexed (10 min more) before the HPLC-HRMS analysis.

For synthetic faeces preparation, baker’s yeast was purchased from Royal (Madrid, Spain), cellulose powder, potassium chloride, and oleic acid were obtained from Sigma-Aldrich (Steinheim, Germany), psyllium husk was acquired from Natureen (Magescq, France), sodium chloride was obtained from Carlo Erba Reagents, calcium chloride and ethanol ≥ 99.5% were purchased from Merck, and miso paste was obtained from HeldenPilz GmbH (Teuchern, Germany). Hydrogen peroxide (30%) was supplied by Fisher Scientific (Pittsburgh, PA, USA), iron (III) sulphate hydrate (97%) used as the catalyst for Fenton’s reagent preparation was purchased from Sigma-Aldrich (Steinheim, Germany), potassium hydroxide (85%) and nitric acid (69%) were acquired from ITW Reagents (Barcelona, Spain).

### 3.2. Preparation of Polyethylene Nanoparticles (PE-NPs)

PE-NPs were prepared by two different procedures: (a) solvent–antisolvent precipitation; and (b) oil-in-water (O/W) emulsification and precipitation within emulsion droplets. The first procedure was based on the precipitation method proposed by Tanaka et al. [19,26]. Briefly, PE was solubilized in toluene at 110 °C, using a reflux system with a condenser to prevent toluene evaporation, and subsequently precipitated by diluting 4 mL of the solution into 100 mL of DMSO at 110 °C. After mixing, the dispersion was cooled down to room temperature, and DMSO was removed through successive washing and filtration processes through dialysis membranes (Servapor dialysis tubing (SERVA, Heidelberg, Germany), MWCO 12,000–14,000, 25 mm flat width). Specifically, the dispersion was divided into several dialysis bags (~25 mL each), and they were dialyzed in batches of two bags against 1 L of Milli-Q water. This purification process consisted of 10 consecutive washing cycles, with each cycle lasting a minimum of 6 h. In summary, toluene dissolved PE, whereas DMSO, being miscible with toluene, acted as an antisolvent that triggered PE precipitation. The scheme of this process is shown in the Supplementary Materials (Figure S1). The second procedure, based on oil-in-water (O/W) emulsification, was adapted from the method described by Merdy et al. [20]. In this approach, the polymer was dissolved in an appropriate organic solvent, O/W emulsions were formed, and PE was subsequently precipitated within the emulsion droplets by lowering the temperature. First, PE was dissolved at various concentrations in 10 g of toluene containing 20 mg of Tween^®^ 80. The mixture was heated overnight at 110 °C under a reflux system for approximately 17 h to ensure complete dissolution of PE. Subsequently, 12 g of a 3.5 g/L NaCl solution were added at 80 °C, and the mixture was vigorously agitated for 180 s with an Ultra-Turrax homogenizer (IKA T18 Digital (IKA, Staufen, Germany), equipped with an S25-10G rotor–stator dispersion unit) at 20,000 rpm, while maintaining the temperature at 80 °C. This agitation led to the formation of a toluene-in-water emulsion. The droplet size was further reduced by ultrasonication at 80 °C to obtain submicron emulsions. PE NPs were then precipitated within the emulsion droplets by cooling the system to approximately 0 °C. The resulting NPs were purified by dialysis, using the Servapor membranes described before, first in ethanol (ten cycles, minimum of 6 h per cycle) and then in water (ten cycles, minimum of 6 h per cycle), monitoring the conductivity until it dropped to 2 µS/cm or less. Final PE concentrations were calculated under the assumptions that the NPs could neither cross nor adsorb onto the dialysis membrane, and that any volume change during dialysis was also negligible. The process scheme is shown in the Supplementary Materials (Figure S2). Although this preparation method produces NPs with relatively high polydispersity, it was selected because the Tween^®^ 80 surfactant, which is edible and not cytotoxic, remains adsorbed on the NP surface and provides higher colloidal stability than the NPs prepared by solvent–antisolvent precipitation.

### 3.3. Characterisation of NPs

***Scanning electron microscopy (SEM)***. A Hitachi TM-4000 Plus II desktop scanning electron microscope (Hitachi, Tokyo, Japan) was used. Samples were dried on a conductive adhesive tape placed on the sample holder, and the instrument was operated at 15 kV.

***Transmission electron microscopy (TEM)***. The characterisation of NPs was performed by TEM using a JEOL JEM-1010 microscope (JEOL, Tokyo, Japan) operating at 80 kV and equipped with a Gatan Orius CCD camera (Gatan, Pleasanton, CA, USA). Negative staining with 2 wt% uranyl acetate was applied to samples placed on Formvar/carbon film (Cu F/C 200 mesh)-coated copper grids. Prior to sample deposition, the grids were UV-treated to enhance film adhesion and increase surface hydrophilicity.

***Dynamic light scattering (DLS) determinations***. The hydrodynamic diameter of the particles was determined by DLS using a 3D DLS spectrometer (LS Instruments, Fribourg, Switzerland) equipped with a 20 mW He–Ne laser (λ = 632.8 nm). The instrument records the scattered intensity of two simultaneous incident beams and cross-correlates the signals to suppress multiple scattering. Samples were measured in 5 mm cylindrical borosilicate tubes at 25 °C, immersed in a decalin bath to match the refractive index of glass. DLS measurements were performed at a scattering angle of 90°. The decay rate and translational diffusion coefficient were calculated using the CONTIN algorithm, which was preferred over the Cumulant method as CONTIN assumes multimodal distributions and thus provides more reliable results for polydisperse samples. The hydrodynamic radius was calculated from the diffusion coefficient using the well-known Stokes–Einstein equation. All measurements were performed in triplicate, and the average particle size and experimental error were reported as the mean ± standard deviation.

### 3.4. Synthetic Faeces

To ensure faecal material without plastic contamination, and where no reference materials were available, synthetic faeces were prepared following the procedure described by Penn et al. [27] and using the reagents and compositions summarised in Table S1 of the Supplementary Materials section. Once the components for the synthetic material were added and homogenised, HPLC-grade water was introduced to achieve a final water content of 65%. Finally, the mixture was held at room temperature for 2 h. In Figure S6 of the Supplementary Materials section, a picture of the synthetic faeces is presented.

### 3.5. Sample Extraction Processes

***Fenton protocol***—A total of 75 mL of reagent was added to 0.5 g of ground and lyophilized sample. The mixture was left to react overnight at 23 °C. Then, the mixture was gently stirred with a glass rod and left to settle for 5 min to allow the NPLs to float. Half of the volume was filtered through 0.7 µm glass microfiber filters facilitated by vacuum pump. The remaining half-volume was centrifuged at 3500 rpm for 15 min, and the supernatant was filtered through the same filter. Subsequently, 25 mL of nitric acid was added directly onto the filter and left for 15 min without applying vacuum. After this period, the vacuum pump was reactivated, and the filter was rinsed with HPLC-grade water in three successive washes to neutralise the acid. Finally, a few drops of ethanol were added to accelerate the drying process. The filters were then left to dry overnight in an oven. Then, the filters were extracted by means of solid–liquid extraction using 10 mL of toluene in an ultrasonic bath for 15 min. The supernatant was collected and two more extractions of the filter with toluene were repeated. The final volume was evaporated under a gentle stream of nitrogen and transferred to a LC vial. This vial was further evaporated to dryness and reconstituted with 1 mL of toluene. The final extracts were kept at −20 °C before injection.

### 3.6. Instrumental Analysis

The analysis was carried out by size-exclusion liquid chromatography (Acquity High Performance, Waters Corporation, Milford, MA, USA) coupled with high-resolution mass spectrometry (Q-Exactive Orbitrap™, Thermo Fisher Scientific, San Jose, CA, USA) equipped with an atmospheric-pressure photoionization (APPI) source (LC(SEC)-APPI(-)-HRMS). The chromatographic separation of the extracts was achieved with an ACQUITY APC™ XT column (45 Å pore size, 1.7 μm particle size, 4.6 mm id, 150 mm length) from Waters (Milford, MA, USA). The column was maintained at 30 °C, and the mobile phase consisted of 100% toluene which was also used as a dopant agent for the APPI source. The injection volume was 10 µL and the flow rate was set to 0.5 mL/min.

The optimal APPI source conditions were as follows: sheath gas at 60 a.u., auxiliary gas at 35 a.u., and S-lens RF at 100 a.u. The capillary and probe temperatures were maintained at 400 °C as described elsewhere [21]. The acquisition was done in full scan mode with a mass range of 500–3000 *m*/*z* and resolution of 17,000 FWHM. The entire system was controlled by Xcalibur 4.1 software.

Data processing was performed with Xcalibur™ Qual Browser 4.1 software (Thermo Fisher Scientific, San Jose, CA, USA). The peak area of the most intense ion corresponding to the common loss of monomers for each polymer was used for the quantification of the compounds.

One of the main challenges in MS analysis of polymers is data mining, as mass spectra are often highly complex due to the composition of technical polymer blends, isotope distributions, adduct formation, multiply charged ions, and fragment ion series [28]. For this reason, Kendrick Mass Defect (KMD) analysis was applied, following the approach proposed by Sato et al. [29].

The *m/z* values obtained by LC-HRMS were converted to Kendrick Mass (KM) according to Equation (2). The Nominal Kendrick Mass (NKM) and the Corrected Nominal Kendrick Mass (CNKM) were calculated according to Equations (3)–(5):

(2) $$\mathrm{KM}\;(\mathrm{ion})\;=\;\frac{m}{z}\;\left(ion\right).\;\frac{round\;\frac{R}{X}}{R/X!}$$

NKM (*ion*) = round (KM (*ion*)) (3)

ΔKMD (*ion*) = NKM (*ion*) − KM (*ion*) (4)

CNKM (ion) = NKM (*ion*)-ceiling (NKM(*ion*) − *m*/*z* (*ion*)) (5)

In these equations, R represents the mass of the repeat unit and X is a positive integer used as the base unit for the calculation of KM (*ion*). In standard KMD analysis, X = 1. However, the concept of a fractional base unit was introduced by Fouquet et al. [28] to improve the resolution of KMD plots. In this study, this approach was applied to achieve a clear separation of ^12^C and ^13^C isotope distributions of PE and PP for each adduct detected by LC-HRMS.

The KMD analysis was used to facilitate the interpretation of the complex LC-HRMS spectra obtained for PE and PP oligomers. The Kendrick Mass scale was calculated using the repeat unit of each polymer as the reference. For PE, the repeat unit is C_2_H_4_ (exact mass 28.03130 Da), whereas for PP it is C_3_H_6_ (exact mass 42.04695 Da). In standard KMD analysis, measured *m/z* values are scaled using the ratio between the nominal and exact mass of the repeat unit. However, polymer spectra often contain overlapping oligomer series, adduct distributions, and isotope patterns, which complicates interpretation.

To improve plot resolution, the repeat unit was multiplied by an integer factor (X) prior to the Kendrick transformation. Increasing X reduces the spacing between KMD and enhances the separation of ^12^C/^13^C isotope envelopes and different adduct series. In this study, X = 102 provided optimal visualisation, allowing clear alignment of the PE and PP oligomer series and resolution of their isotope patterns for each detected adduct. This approach significantly improved the interpretability of KMD plots and facilitated polymer identification in complex environmental samples.

For polymers analysed by LC-HRMS, high-resolution mass spectra combined with KMD analysis provide the minimum identification points, since the retention time in the APC column depends primarily on molecular weight rather than polymer type.

Because the molecular weight of polymers present in samples may differ from that of the calibration standards, a concentration conversion was required. The area of the most intense peak in the mass spectral profile was used for quantification when the polymer MW differed from 1200 Da. The concentration obtained from the calibration curve was converted to an equivalent polymer concentration according to Equation (6). The equivalent factor (Feq) depends on the number of monomers present in the standard (*n. mon*.) and in the sample (*n. mon*.) (Equation (7)). The *n. mon* are the rounded values obtained by dividing the mass of the highest peak of the polymer’s profile by the mass of monomer.

(6) $${\left[\mathrm{Pol}\right]}_{\mathrm{eq}}=\frac{\left[Pol\right]cc}{Feq}$$

(7) $$Feq=\left(n.\;mon\right)sample/\left(n.mon\right)std$$

### 3.7. Spiking Experiments and Matrix-Matched Calibration Curves

The instrumental quality parameters were calculated using ten points calibration curves in toluene working with two ranges: from 0.005 to 50 µg/L and from 50 to 1000 µg/L. Instrumental linearity and ranges were estimated using linear regressions with good results in both ranges (R^2^ > 0.995). Instrumental precision (n = 5), expressed as the relative standard deviation (RSD%) of intra-day and inter-day repeatability of three different concentration levels (5, 50, and 500 µg/L), was evaluated. In all the cases, precision was lower than 5% and 25% for intra- and inter-day evaluations, respectively. The ILOD was estimated from the injection (10 µL) of the lowest calibration curve point (0.5 pg).

To assess the best extraction approach, PE and PP-NPs were used to fortify the synthetic faeces at a concentration of 143 µg/kg. The samples were spiked individually with each polymer. The samples were left at 4 °C overnight to reach equilibrium and then were freeze-dried and extracted by the different tested methods, working in triplicate. Procedural blanks were included to monitor potential contamination and ensure analytical reliability.

Once the extraction approach was chosen, validation of the methodology (Fenton’s reagent method) was performed according to the protocol described above, fortifying the synthetic faeces at concentrations ranging from 1.4 to 71 µg/kg, working in triplicate.

To evaluate the analytical performance of the preparation method, a matrix-matched calibration curve was prepared. A blank matrix was extracted by the Fenton’s reagent method due to its superior performance. The blank extracts were then fortified with suspensions of PE and PP-NPs in the range from 14 ng/kg to 143 µg/kg by serial dilutions (1:10).

Recovery experiments with the synthetic material were carried out by spiking it from 1.4 to 71 µg/kg, conducted according to the 2002/657/EC European Commission Decision [30]. Linearity, intra-day repeatability, and method accuracy were also evaluated after spiking.

The MLOD and MLOQ were calculated using the following equations:

MLOD = 3.3 s/b(8)

MLOQ = 10 s/b (9)

where b is the slope and s is the standard deviation of the signal determined from the standard deviation of the intercept in the linear regression of the polymer calibration curves

### 3.8. Real Samples Analysis

To assess the performance of the analytical method and the sampling approach, five samples from different volunteers were analysed. Each volunteer was provided with two stainless steel containers: one for faeces collection and a second container that was transported alongside it to serve as a contamination blank. Both containers were opened and closed simultaneously, and after sample collection, they were first covered with aluminium foil and then sealed with silicone lids.

All samples were transported to the laboratory on the same day of sampling, frozen at −20 °C, lyophilised, homogenised, and extracted according to the protocol described in Section 3.7. Analysis was carried out as described in Section 3.8.

The blank containers were rinsed with toluene (3 × 20 mL). The combined 60 mL extract was ultrasonicated and reduced in volume to less than 1 mL under a nitrogen stream, transferred to an LC vial, and finally adjusted to a volume of 1 mL with toluene. The blank extract corresponding to each volunteer was subtracted from the respective sample result.

### 3.9. Quality Assurance and Quality Control (QA/QC) of the Analytical Method

To minimise background contamination, all sample pre-treatment and manipulation were conducted under a laminar airflow bench in a plastic-free laboratory. Personnel wore cotton lab coats throughout the procedures. Glassware or stainless steel was used for all manipulations whenever feasible.

To monitor potential external contamination, procedural blanks consisting of ultra-pure HPLC-grade toluene were included in each extraction batch. Additionally, LC vial caps were replaced with aluminium foil to prevent contamination during LC-HRMS analysis. No MNPLs were detected in any of the toluene procedural blanks. In the case of synthetic faecal material, the background was obtained and subtracted from the samples, as well as from sampling blanks, prior to quantification by the Xcalibur Qual Browser software. Instrumental cross-contamination was evaluated by injecting solvent blanks, toluene for polymers, between each sample injection.

## 4. Conclusions

PE nanometric particles were synthesised following the emulsion–precipitation method described by Merdy. The resulting suspensions contained particles with mean hydrodynamic diameters of 340 ± 61 nm and 259 ± 66 nm, depending on the polymer concentration (500 and 136 mg/L), with PDIs of 0.35 and 0.36, respectively.

Synthetic faeces were spiked at different levels, and an extraction method for nanoplastic-like particles was developed and characterised for two of the most commonly reported polymers in human faeces: PE and PP.

The method requires only a small amount of faecal material (0.5 g), and the use of Fenton reagent yields moderate recoveries ranging from 51% to 66%. Good intra-day and inter-day precision was obtained, with relative standard deviations (RSDs) always below 25%. However, the method was affected by strong matrix effects, necessitating matrix-matched calibration curves to compensate.

NPLs were identified and semi-quantified using SEC(LC)-APPI(-)-HRMS, combined with the Kendrick Mass Defect approach to obtain sufficient identification points. The final optimised approach showed good sensitivity, with MLODs of 0.015 and 0.025 ng/gd.w for PE and PP, and MLOQs of 0.058 and 0.083 ng/gd.w, respectively. This analytical approach presents the main advantages of mass quantification of nanoparticles: high sensitivity compared with other spectrometric approaches, such as Py-GC-HRMS, and no particle size limitation, as occurs with spectrometric approaches. The method was applied to five real faecal samples as a proof of concept to demonstrate its feasibility and applicability, but given the limited number of samples, the results should not be interpreted as a full quantitative validation in authentic matrices or as a representative biomonitoring study. Finally, the results showed PE was detected in two of the analysed samples, whereas PP was not observed.

## Figures and Tables

**Table 1:** DLS parameters obtained for PE-NPs prepared with both methods.

	Emulsion Method		Antisolvent Method
Concentration (mg/L)	500	136	39
Hydrodynamic diameter (nm)	340 ± 61	259 ± 66	390 ± 50
PDI	0.35	0.36	0.47

**Table 2:** Quality parameters.

Polymer	PP		PE	
**Linearity**	0.005 to 50 µg/L		0.005 to 50 µg/L	
	50 to 1000 µg/L		50 to 1000 µg/L	
**Matrix effect**	31%		−52%	
**Recovery (%) at different spiking levels**	**Spiking level**	**Recovery (%RSD)**	**Spiking level**	**Recovery (%RSD)**
	71.4 µg/kg	60.93% (8.6)	71.4 µg/kg	65.75 (7.8)
	14.3 µg/kg	59.2% (10.2)	14.3 µg/kg	54.68 (24.7)
	1.4 µg/kg	61.1% (20.1)	1.4 µg/kg	55.34 (10.1)
**MLOD**	0.025 µg/kg		0.015 µg/kg	
**MLOQ**	0.083 µg/kg		0.058 µg/kg	
**Intra-day precision (%RSD)**	20.1		10.1	

**Table 3:** Results in real samples.

		NEGATIVE APPI						
Dry Weight (mg)	Samples	*m*/*z* Intense Peak	Most Intense Peak Area	*m*/*z* Range of the Polymer	tr (min)	Concentration in Vial (ng/mL)	n	Real Concentration eq (µg/g)
	Toluene		nd					nd
	Blank		nd					nd
1082.7	1	817.63	2,136,354,135	650–1100	2.19	35,329	29.2	34.03
1199.1	2	1539.04	9,930,474,141	1400–1650	2.77	164,341	55.0	269.04
1470.3	3		nd					nd
2046.3	4		nd					nd
1920.1	5		nd					nd

## Data Availability

The original contributions presented in this study are included in the article/Supplementary Materials. Further inquiries can be directed at the corresponding authors.

## Supplementary Materials

The following supporting information can be downloaded at: https://www.mdpi.com/article/10.3390/molecules31111947/s1, Table S1. Chemicals and materials used for simulant preparation. Figure S1. Scheme of the procedure used for PE nanoparticle preparation. Image adapted from Tanaka et al. [19]. Figure S2. Schematic overview of the preparation of PE nanoparticles. Image adapted from Merdy et al. [20]. Figure S3. Transmission electron microscopy (TEM) images of the PE samples obtained by the solvent-antisolvent precipitation method, at 39 ppm, (a) and (b), and 4 ppm, (c). Figure S4. TEM images of NPs obtained using Merdy’s emulsion method, with 136 (a) and 500 ppm PE concentrations. Both scale bars indicate 200 nm. Figure S5. Example of an extracted ion chromatogram (on top) of the *m*/*z* 1531–1551 and the corresponding mass spectrum for the retention time 2.76 (down) of a PE in a real sample. Figure S6. Synthetic faecal material.
