# Supplementary material for: Optimisation and Validation of a Quantitative Method for the Analysis of Polymers of Nanoplastics in Human Faeces
Source: Molecules. 2026 Jun 4;31(11):1947. doi: 10.3390/molecules31111947 (PMC13257795; doi:10.3390/molecules31111947)
Supplement: Supplementary file 1 [file molecules-31-01947-s001.zip › molecules-4315755-supplementary.pdf]

SUPPORTING INFORMATION

Optimization and validation of a quantitative method for the analysis of polymers of nanoplastics in human faeces

Eloy Torres[1], Mireia Obon[2,3,4], Víctor Moreno[2,3,4,5], Ferran Moratalla[2,3,4,5], Jordi Esquena[5], Marta Llorca[1], Marinella Farré[1,*].

[1]ON-HEALTH group, Institute for Environmental Assessment and Water Research (IDAEA-CSIC) Barcelona, Spain.
2ONCOBELL Program, Bellvitge Biomedical Research Institute (IDIBELL), L'Hospitalet de Llobregat, 08908 Barcelona, Spain
3Unit of Biomarkers and Susceptibility (UBS), Oncology Data Analytics Program (ODAP), Catalan Institute of Oncology (ICO), L'Hospitalet del Llobregat, 08908 Barcelona, Spain
4Consortium for Biomedical Research in Epidemiology and Public Health (CIBERESP), 28029 Madrid, Spain
5Department of Clinical Sciences, Faculty of Medicine and health Sciences and Universitat de Barcelona Institute of Complex Systems (UBICS), University of Barcelona (UB), L'Hospitalet de Llobregat, 08908 Barcelona, Spain
[6]Institute for Advanced Chemistry of Catalonia (IQAC-CSIC), and Networking Research Center on Bioengineering, Biomaterials and Nanomedicine (CIBER-BBN), Barcelona, Spain.

Table S1. Chemicals and materials used for simulant preparation.

Component	Composition of solid content (wt%)
Baker's yeast	30
Cellulose	10
Psyllium husk	17.5
NaCl	2
KCl	2
$CaCl_2$	1
Oleic acid	20
Miso paste	17.5

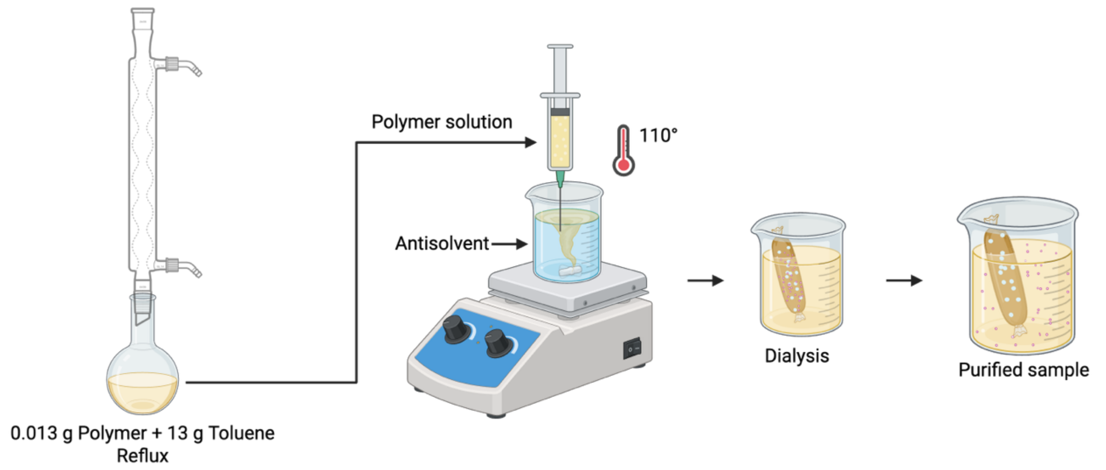

Figure S1. Scheme of the procedure used for PE nanoparticle preparation. Image adapted from Tanaka et al. [19].

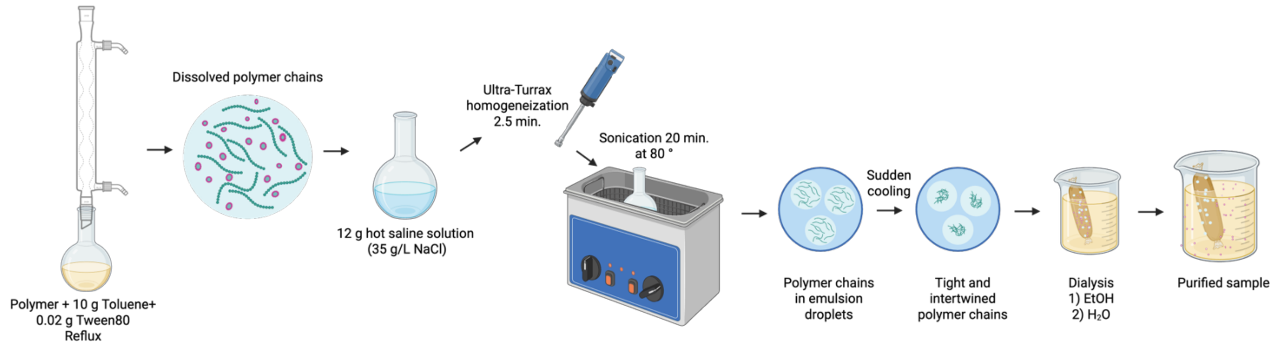

Figure S2. Schematic overview of the preparation of PE nanoparticles. Image adapted from Merdy et al. [20].

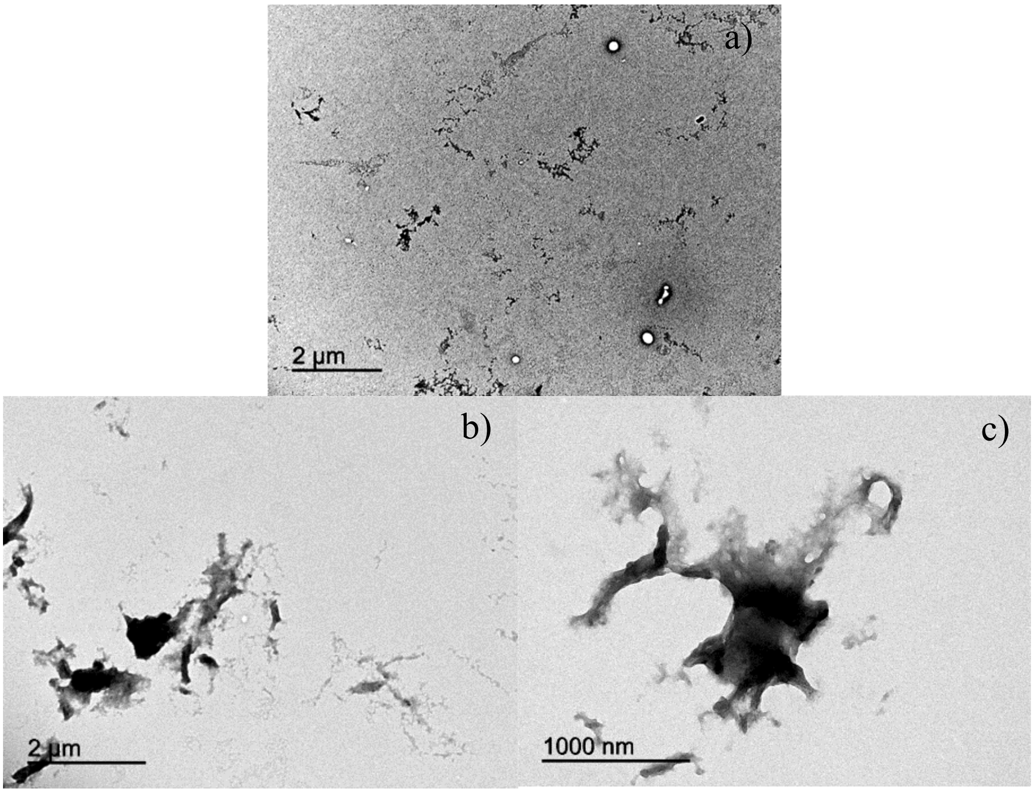

Figure S3. Transmission electron microscopy (TEM) images of the PE samples obtained by the solvent-antisolvent precipitation method, at 39 ppm, (a) and (b), and 4 ppm, (c).

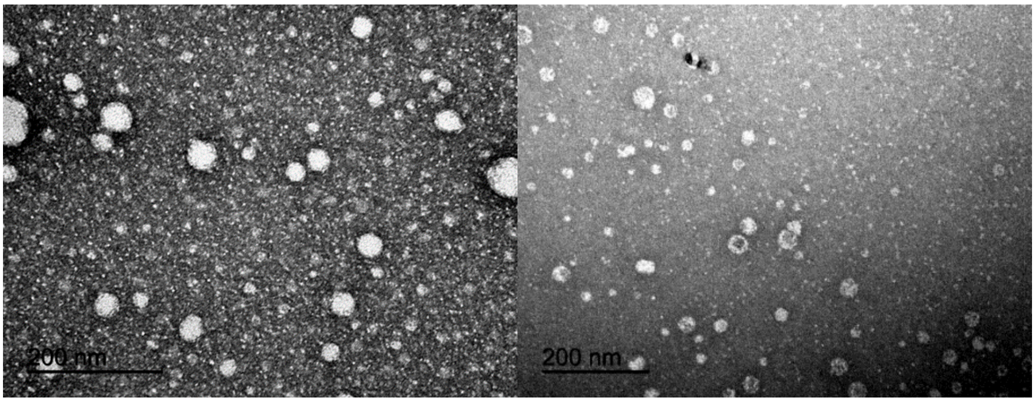

Figure S4. TEM images of NPs obtained using Merdy's emulsion method, with 136 (a) and 500 ppm PE concentrations. Both scale bars indicate 200 nm.

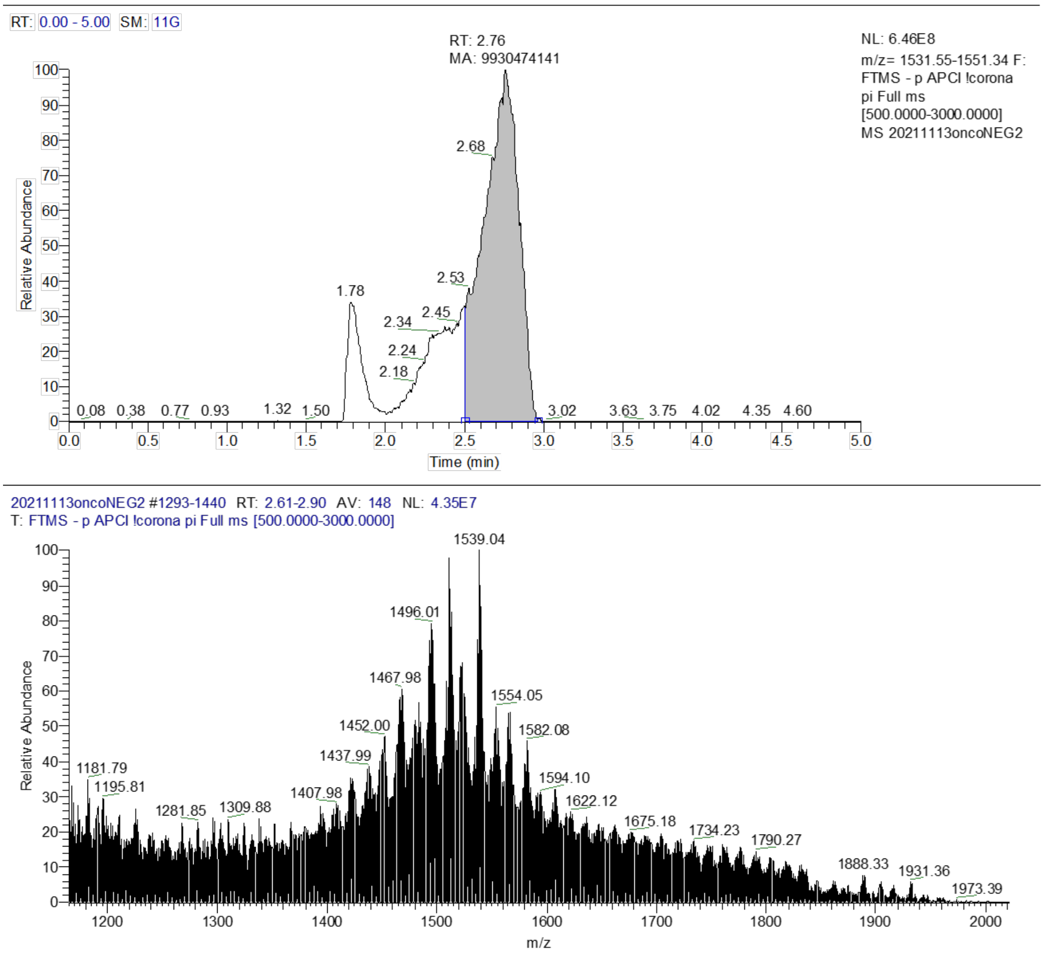

Figure S5. Example of an extracted ion chromatogram (on top) of the m/z 1531-1551and the corresponding mass spectrum for the retention time 2.76 (down) of a PE in a real sample.

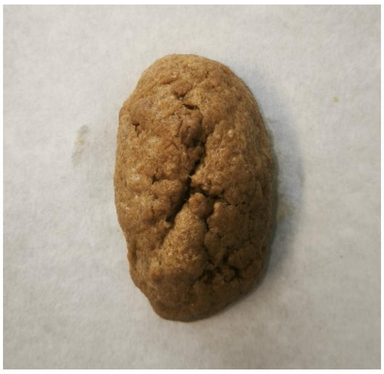

Figure S6. Synthetic faecal material.
